# Application of PK/PD Modeling in Veterinary Field: Dose Optimization and Drug Resistance Prediction

**DOI:** 10.1155/2016/5465678

**Published:** 2016-02-16

**Authors:** Ijaz Ahmad, Lingli Huang, Haihong Hao, Pascal Sanders, Zonghui Yuan

**Affiliations:** ^1^National Reference Laboratory of Veterinary Drug Residues (HZAU) and MAO Key Laboratory for Detection of Veterinary Drug Residues, Huazhong Agricultural University, Wuhan, Hubei 430070, China; ^2^The University of Agriculture Peshawar, Khyber Pakhtunkhwa 25130, Pakistan; ^3^MOA Laboratory for Risk Assessment of Quality and Safety of Livestock and Poultry Products, Huazhong Agricultural University, Wuhan, Hubei 430070, China; ^4^Laboratory of Fougères, French Agency for Food, Environmental and Occupational Safety, 94701 Maisons-Alfort Cedex, France

## Abstract

Among veterinary drugs, antibiotics are frequently used. The true mean of antibiotic treatment is to administer dose of drug that will have enough high possibility of attaining the preferred curative effect, with adequately low chance of concentration associated toxicity. Rising of antibacterial resistance and lack of novel antibiotic is a global crisis; therefore there is an urgent need to overcome this problem. Inappropriate antibiotic selection, group treatment, and suboptimal dosing are mostly responsible for the mentioned problem. One approach to minimizing the antibacterial resistance is to optimize the dosage regimen. PK/PD model is important realm to be used for that purpose from several years. PK/PD model describes the relationship between drug potency, microorganism exposed to drug, and the effect observed. Proper use of the most modern PK/PD modeling approaches in veterinary medicine can optimize the dosage for patient, which in turn reduce toxicity and reduce the emergence of resistance. The aim of this review is to look at the existing state and application of PK/PD in veterinary medicine based on* in vitro*,* in vivo*, healthy, and disease model.

## 1. Introduction

Antimicrobial drugs are the most frequently used veterinary drugs [1–3]. Worldwide, animals are frequently treated with antimicrobials to cure and prevent disease as well as promote growth [1, 4–7]. Prevention and treatment of bacterial disease of animals are medical indications of antimicrobial treatment while growth promotion obtained by oral administration at low dose during long period is considered as a zootechnical use of antimicrobial with economical outcomes. Antimicrobial at subtherapeutic levels would change the feed conversion activity of bacteria in the gut, resulting in weight gain benefits. European Union has stopped the use of all antimicrobial feed additives with this zootechnical claim between 1997 and 2006 [8]. In 2012 the U.S. Food and Drug Administration implemented a strategy to discourage the use of antibiotics for production purposes by imposing the pharmaceutical industry to remove growth promoters from approved product and by requiring veterinary oversight for drugs used in food-producing animals.

For each use of antimicrobials, it is considered that antimicrobial effect creates a selective pressure in the treated animal and its environment [9]. Indeed, during treatment period, high antimicrobial concentrations are obtained at site of action to kill or control development of pathogens, simultaneously; commensal bacteria of the microbiota (gut, skin, mouth, etc.) are exposed. Moreover, the active drug will be excreted and contaminated environment where environmental bacteria will be exposed to subinhibitory concentrations [10]. Today the main risk considered by veterinary guidelines (Codex Alimentarius) [11] (VICH 2003) [12] is about the development of antimicrobial resistance for zoonotic bacteria (*Salmonella enterica*,* Campylobacter* sp.) and commensals (*E. coli*,* Enterococcus* sp.) [13]. Threat of development of resistance at the consumer level through exposure to residue is assessed according to guidelines for industry (VICH 2007) to derive a microbiological acceptable daily intake [14].

Development of antimicrobial resistant bacteria is a complex process combining selection and spread in different compartments (human, animal, and environment) interconnected from epidemiological and ecological perspectives [15].

Risk management of antimicrobial resistant bacteria requires numerous actions in veterinary medicine. (1) Bacterial disease in animal production must be prevented by good farming practices, biosecurity, and prevention through vaccination instead of preventive use of antimicrobials [16]. (2) Antimicrobials must be used to cure bacterial infections which require an improvement of rapid accurate diagnosis at farm levels. (3) Optimal dosage must be used to maximize drug efficacy against target pathogens and minimize exposure of commensal flora [17]. (4) Spread of antimicrobial resistance carried by genes and bacteria must be limited and controlled through hygiene and disinfection [16]. (5) Human health risks attributable to veterinary usage must be assessed and maintained as low as reasonably achievable. We also need to increase awareness regarding antibacterial use and optimize the dosage regimen of antibacterial.

Old and new drugs are being used in different parts of the world with a wide range of regulatory and management context. As human growth in the next 30 years will lead to an increase of animal production, one of the most important challenges for veterinarians, veterinary pharmaceutical companies, and animal and food producers will be to maintain an efficient and safe usage of antimicrobial drugs as well as acceptation of their methods of production by consumers without misperception of risk associated with veterinary antimicrobial usage [18]. The purpose of this review is to present and discuss the use of pharmacokinetics (PK) and pharmacodynamics (PD) for antimicrobial drugs and their combination (PK/PD) in the rejuvenating of old drugs and development of new ones in veterinary medicine. It will first review the PK/PD indices used to optimize dose against a target pathogen and in the second part discuss the challenge for PK/PD model to assess risk of antimicrobial development for the target pathogens and the commensal bacteria.

## 2. General Principles and Methodology of PK/PD

**Figure 1:**
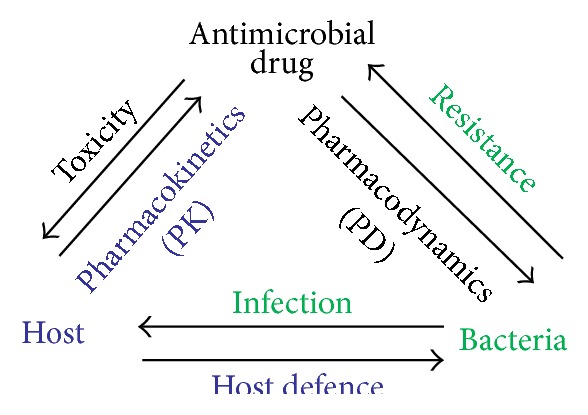
Triangular relationship between an antimicrobial drug, host, and bacteria during a treatment.

Pharmacological modeling study deals with pharmacokinetics (PK) and pharmacodynamics (PD). PK describes “what the body does to a drug,” that is, how it is absorbed, distributed, and eliminated through metabolism and excretion, and PD describes “what a drug does to the body,” that is, how it interacts with receptors and their signaling pathways up to the whole body level. The time course of drug concentration and its effects is modeling outcomes. Altogether, PK-PD models provide a powerful tool to connect dosage regimens to clinical effects and vice versa [19]. The PK/PD model describes the relationship of potency, exposure of microorganism, and the effect of antimicrobial agents (Figure 1).

Pharmacokinetic/pharmacodynamic (PK/PD) modeling approaches used to establish a dosage schedule best suitable for promoting the eradication of bacteria, as a result reducing the hazard of determined carrier status and the progress of resistance, have been used in the veterinary field [20, 21]. The regulatory agencies also recommend that PK/PD relationship investigations are included in drug development procedure (EMA and FDA Guidelines) [22, 23]. However, most of the antibacterial drugs nowadays in the market were developed several decades ago. For these agents the dosing regimens are optimized usually on the base of point estimates (in terms of the MIC) and observed clinical efficacy. For example, colistin (polymyxin) antibiotics are increasingly used, but their old development was not on the basis of rigorous drug evaluation method [24], while on the other hand, today, advancement in modern technology allows us to use more computer based techniques for the investigation of complex PK-PD relationships. Progressively, simulation-based techniques are mostly used in therapeutic areas and made available for a quantitative description of the time course of drug effects, which have great ability for achieving a more optimal drug therapy [25–28] that can also reorganize the development of drug and help in critical decisions. The decisions include designing and planning of the most favorable dosing regimen in clinical trials [29]. The research on PK study is highly established, and many software programs are available for the determination of pharmacokinetics parameters such as volume of drug distribution, clearance, and area under the concentration time curve and dosage simulations. On the other hand, pharmacodynamics parameters have not been widely characterized; however, frequently, an adapted maximum effect (*E*_max_) model can be used and EC_50_ (concentration at half-maximum effect) can be calculated to exemplify the exposure/response correlation [30]. The PD study linked drug exposure to the effect observed after drug administration [31].

## 3. PK/PD Index

**Figure 2:**
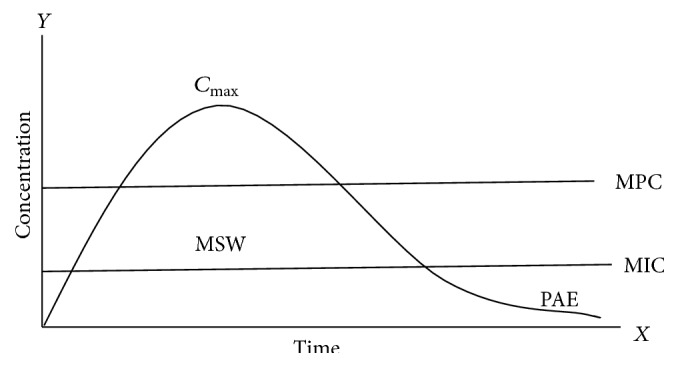
Antimicrobial PK and PD parameters in relation to MIC; the most useful PK parameters are the area under the plasma concentration time curve (AUC) from 0 times to 24 h, the maximum plasma concentration (*C*_max_) achieved, and time (*T*) during which concentration exceeds a defined threshold. The most useful PD parameter is the minimum inhibitory concentration (MIC).

A valuable strategy of dosing for anti-infective requires a comprehensive understanding of the complex connections between microbe, drug, and the host immune system. As shown in Figure 1, pharmacokinetic and pharmacodynamic (PK-PD) modeling has been developed to simplify these relationships to assist the dose optimization and dose selection of antimicrobial agents [32]. The potency and efficacy of antimicrobial drugs are generally defined by the MIC, minimum bactericidal concentration (MBC), and PD parameters which are determined* in vitro*. For fast growing organisms, the MIC is defined as the lowest drug concentration that leads to no visible growth of a bacterial strain after incubation of 24 h under approved conditions. The MBC is the concentration that decreases the bacterial population up to 99.9% of a given organism after 24 h of exposure. Other PD descriptors can be derived from time kill curve studies and provide more information regarding extent of killing at different concentration and time [33] (see (4) in Section 6). Antimicrobial effect on bacteria is classified into two types of relationships: concentration-dependent and time-dependent. For concentration-dependent drug, bacterial killing effect increases with concentrations in the range of concentrations obtained at the target site. For time-dependent drug, bacterial killing effect reaches a maximal value even if concentration increases, so effect is dependent on time of exposure (Figure 2, Table 1).

To determine pharmacodynamic properties of antimicrobial drugs such as MIC, use of reference method is recommended [34]. Method used to obtain time kill curves must be described or performed according to published technical recommendations. This point is critical to bridge the dose derived from PK/PD preclinical studies with the use of MIC for antimicrobial susceptibility testing for diagnostic and antimicrobial resistance monitoring [35, 36].

The concept of PK/PD indices to optimize dose is derived from the pharmacoepidemiological analysis of results of randomized clinical trials, monitoring antimicrobial efficacy in hospital ward with individual adjusted therapy. By comparison with experimental models results, the predictability of clinical outcomes of the different indices was validated by observation in human medicine [37]. It was one of the major types of progress on the last decade in human medicine because it established objective values to expect a favorable outcome in patient populations. Basically a relationship exists between the efficacy of an antimicrobial against bacteria and the concentration-time profile and a prediction of the likelihood of successful treatment can be made [36]. It was determined that a relationship between PK/PD index and clinical outcomes in different kind of infection in humans can be established. Moreover, the relationship between a PK/PD index and response to treatment allows for the definition of a pharmacodynamics target (PDT). The PDT is the minimum value of the PK/PD index that is aimed at treating patients and is based on preclinical and clinical drug/microorganism exposure-response relationships. The PDT ideally is the PK/PD index value that ensures a high probability of successful treatment [36].

To establish PK/PD index, the most valuable pharmacokinetics parameters include the area under the plasma concentration time curve (AUC) from 0 times to 24 h, the drug peak concentration in plasma (*C*_max_) achieved, and time (*T* > MIC) during which concentration goes beyond the minimum inhibitory concentration (MIC) [38, 39]. The three most used PK-PD indices are the duration of time during which drug concentration remains above the MIC (*T* > MIC), the ratio of the peak drug concentration to the MIC (*C*_max_ : MIC), and ratio of the area under the concentration time curve at 24 h to the MIC (AUC_0–24_ : MIC) which are shown in Figure 2 [40, 41]. Some of the important PK, PD, and PK/PD indices definitions and their units are summarized in Table 2.

It is recommended to establish the pharmacokinetic parameters with the unbound (free) fraction of the drug as it is usually proportional to interstitial fluid that surrounds the pathogens [42, 43].

In human medicine, some researcher established PDT for different drugs and indications. As discussed by Mouton et al. [36], two methods can be used to establish PDT from clinical trials (classification tree analysis, examination of the full exposure-outcomes relationship) data analyzed by statistical methods described by [40]. It is recommended that both methods suffer from the fact that in many clinical trials there are not enough failures to perform such analyses, particularly for new agents. The PDT is therefore most often derived from preclinical studies, such as studies in animal models or from* in vitro* studies such as hollow fiber infection model. In veterinary medicine, this approach can be used in infection model in a target species or laboratory animals to explore different dosage regimens on the clinical and bacteriological outcomes [46].

### 3.1. Concentration-Dependent

To evaluate the activity of antimicrobial agent for drug that showed concentration-dependent killing the higher the drug concentration, the larger the level of bacterial killing, so the maximum concentration will be the best predictor for efficacy. Fluoroquinolones and aminoglycosides display this type of killing [37]. It has also shown in* in vitro* model that AUC/MIC for fluoroquinolones is best related to the antibacterial effect against* Pseudomonas aeruginosa* and* Streptococcus pneumonia* [47]. The PK/PD surrogates that show association with clinical outcomes for these drugs can be resulting from one of two ratios: *C*_max_/MIC or AUC/MIC. For better clinical cure and bacterial eradication, the AUC/MIC ratio for immunocompetent animals against Gram-positive pathogens should exceed 25–30, while in case of immunocompromised animals the ratio of AUC/MIC should exceed 100–125 against Gram-negative pathogens for fluoroquinolones. In case of concentration-dependent drugs *C*_max_ is the suitable parameter for evaluating the pharmacokinetics of drug that kills bacteria through concentration-dependent mode of action. For that type of drug, the once daily dosing will be the most effective, as long as the drug has sufficient half-life or also has prolonged postantibiotic effect. For aminoglycoside, the once daily dose will be efficient to reach a high *C*_max_/MIC ratio. For this class, this dosage regimen reduces the level of ototoxicity [48]. Balaje et al. [49] investigated PK/PD relationship of enrofloxacin against* Pasteurella multocida* in buffalo calves. For enrofloxacin, the main PK/PD parameters responsible for the efficacy of this drug are *C*_max_/MIC and AUC/MIC [38, 49].

As shown in literature PK/PD model is used for the determination of dosage schedule of marbofloxacin against* Mannheimia haemolytica *causing disease in sheep [50]. These investigations assist in optimizing efficacy of the mentioned drug. PK-PD model experiments with antibacterial were conducted with goats, calves, cows, and dogs [51–54].

### 3.2. Time-Dependent

For time-dependent antibacterial agents, PK/PD indices correlated with efficacy which is the fraction of time of which drug concentration remains above the MIC along a dosing interval. According to experimental study in laboratory animals, human clinical trial, and* in vitro* simulation, *T* > MIC target to reach should be different for different beta-lactam agents, such as for carbapenems (15–25%) compared to penicillin (30–40%) and cephalosporin (40–50%) [55–57]. For time-dependent drugs, there is no difference in activity against both Gram-positive and Gram-negative pathogens. Dose can be optimized to maintain the drug concentration above the MIC of targeted bacteria according to the interval of time between each dose. Cefquinome is time-dependent antibacterial agent and the main PK/PD index responsible for the efficacy of this drug is the time at which the drug concentration is above the MIC. For example, PK/PD integration studies were performed against* Staphylococcus aureus* in calves [58].

The examples of some of the antibacterials and their effect as time- and concentration-dependent according to PK/PD indices are shown in Table 3.

## 4. PK/PD and Clinical Breakpoint

As explained by Mouton et al. [36] to reach a particular PDT in a patient and thereby achieve a high probability of microbiological and clinical cure require an adequate exposure of the bacteria to the antimicrobial agent. The exposure depends on the dose applied and the pharmacokinetics in the patient. Between patients, exposure is function of different pharmacokinetics parameters such as drug bioavailability and clearance. The exposure is also function of the susceptibility of the pathogen (e.g., MIC). An optimal practice of a drug will be to use a dose which is able to reach a pharmacodynamics target for a range of pathogenic bacterial species. The dose can be established according to the bacterial infection targeted by the treatment. Then for a defined dose, it is possible to define a range of MICs with a high probability of cure according to the PDT. From these calculations, MIC with a low probability of cure according to PDT will also be established and a breakpoint based on PK/PD proposed [36].

For different antimicrobials, these approaches based on population pharmacokinetic approach and MIC distribution of pathogens analysis are currently used to assess the potential of a dose to treat a systemic infection by a pathogen. They are used by CLSI and EUCAST to establish a clinical breakpoint. In veterinary medicine, this approach was also applied to establish a clinical breakpoint of few drugs [61].

## 5. PK/PD and Dose

If the distributions of MIC for the different pathogens targeted by the treatment are known, it is also possible to estimate the range of doses necessary to obtain a probability of cure for each pathogen according to the population pharmacokinetics data.

The optimal dosage for drugs whose efficacy can be associated with AUC_0–24_ : MIC ratio can be determined by using the following equation [62]. This equation is helpful to obtain the dose per day [50, 51, 63]:

(1) $$\begin{array}{c} Dose=\frac{\left(AUC_{24}/MIC\right)\cdot MIC\cdot CL}{\text{fu}\cdot F}, \end{array}$$

where AUC_24_/MIC ratio is used for optimal efficacy for a daily treatment; MIC is minimum inhibitory concentration; CL is drug clearance; fu is free fraction of drug in plasma; *F* is bioavailability of drug.

For time dependent drug the PK/PD indices responsible for the efficacy are the time drug concentration remains above the MIC and can be calculated by using the following equation [64]:

(2) $$\begin{array}{c} T>MIC=\ln\left(\;\frac{D}{Vd\cdot MIC}\;\right)\cdot \frac{T1/2\beta }{\ln2}\cdot \frac{100}{t}. \end{array}$$

A weighted AUC (WAUC) has also been useful in dosage optimization [65]. This incorporates the entire time for which the plasma drug concentration exceeds the MIC (3) and can be used for both concentration- and time-dependent drugs. One has

(3) $$\begin{array}{c} WAUC\left(\;h\;\right)=\frac{AUC_{h}\times T>MIC}{MIC{{\left(T>MIC\right)}_{Max}}_{\left(h\right)}}, \end{array}$$

where WAUC_(*h*)_ is area under the concentration time curve weighted for entire time at which plasma drug concentration exceeds MIC (*T* > MIC)_max_ = 24 h [38].

## 6. PK/PD Mathematical Modeling

*In vitro* and* in vivo* models used for the PK/PD integration by mathematical model have some advantages and disadvantages. Semimechanistic PK/PD model combines two submodels: (1) to describe pharmacokinetics in function of drug exposure (dosage regimen) and (2) to describe the natural growth and killing of bacteria versus time and action of antibiotic concentration.* In vitro*, antibiotic effect on the bacterial growth and killing kinetics can be observed in static conditions at defined concentrations or in dynamic conditions mimicking the pharmacokinetic of drugs (time kill curves). Classical pharmacokinetic models are based on virtual compartments (central, peripheral(s)) while physiologically based pharmacokinetic model described tissue distribution according to physiological parameters such as the tissue volume and blood flow in each tissue.

Basically, kill curves observed for one antibiotic concentration *C* can be described by the following equation which is derived from the model proposed [66]:

(4) $$\begin{array}{c} \frac{dB}{dt}=k_{net}\times \left(\;1-\frac{B}{B_{max}}\;\right)\times B-\left(\;\frac{E_{max}\times C^{\gamma }}{{EC}_{50}^{\gamma }+C^{\gamma }}\;\right)\times B, \end{array}$$

where *B* is the number of bacterial cells expressed as cfu/mL, *k*_net_ the net growth rate, *B*_max_ the maximum number of bacteria, *E*_max_ the maximum killing rate, EC_50_ the concentration to reach half of maximal killing rate, and *γ* the steepness. The concentration *C* can be those observed in the central compartment or one calculated for a site of action. A recent review discussed in depth different kinds of models developed recently for some drugs [67].

Semimechanistic PK/PD model developed from* in vitro* data has the capability to simulate the bacterial size at the site of infection in function of time. So there are some software programs being used to simulate different dosage regimens and their effect on the theoretical bacterial cure as well as test the effect of the treatment on different inoculum sizes and bacterial growth. The PK-PD model may therefore limit the need for labor-intensive experiments with dynamic drug concentrations and provide a useful tool for optimizing the dosing regimens and design of future preclinical and clinical studies of antibacterial efficacy.

Disease models provide very important insight about drug efficacy and can be used to explore factors in relation to host response and infection process. Efficacy of antimicrobial drug is also dependent on the start of treatment according to the ongoing infection process in host. Indeed, efficacy of antimicrobials is also dependent on inoculum size at the site of infection as well as the bacteria state and access capacity of drugs to bacterial cells [68]. These are dependent on the host response and bacterial pathogenicity and evolve according to time from an immune acute response to a chronic state with consolidated tissue lesions along the inflammatory process. Some progress must be made to take into account the initial conditions of the treatment as shown experimentally by some works about the relationship between the inoculum size and the development of resistance [69, 70].

## 7. PK/PD and Antimicrobial Resistance

Resistance to antimicrobial drugs can take place through the acquisition of new genes or through point mutation in chromosome [31]. Antibiotic resistance is now at a high priority rank as major public health concern according to WHO, EU, and US risk managers. It is a major problem particularly in the developing countries, where 2nd and 3rd generation antibiotic are not available or high-priced [71]. It is thought that antibacterial resistance is build up quickly in response to the use of antibiotic [1]. Bacterial resistance development is a complex process with different phases of emergence, establishment, increase, and equilibrium leading to extinction or coexistence with susceptible bacteria [72]. Moreover, emergence, establishment, and so forth outside of the treated organisms must be also taken into account to explain the spread and development of antimicrobial resistance in human and animal populations. During treatment, one main cause of selection of resistance is the underexposure of bacterial strains to antibiotics [73, 74].

Appropriate dosing of antibiotic is the key to control or clear bacteria on the site of infection but also to limit antimicrobial resistance. Old and new antimicrobial drugs that are used in veterinary field require more attention in the context of antibiotic resistance. In the early period of development and approval of antimicrobial drugs, they were licensed without proof of efficacy established by randomized clinical trials [75]. Moreover, development of antimicrobial resistance was not an outcome taken into account as a side effect of treatment. During decades, drug formulations and dosing regimen were tested and improved to increase efficacy and reduce side effects (toxicity) from a patient point of view but do not take into account the ecological impact on the microbiota accepted in case of side effects on the intestinal flora [76]. By this way, antibiotic dosage regimen varies over time and between places. Each individual treatment opens a window for selection of antibiotic resistance. After decades of use, many old drugs, widely used, have led to selection of antibiotic resistance in a large range of bacterial species. To obtain a sustainable use of these old drugs, it is necessary to reassess these drugs in the light of our knowledge of the exposure response relationship based on the PK/PD model [73]. The PK/PD indices are mostly used as targets for efficacy in the process of dose selection [28], but it is also necessary to work on the dosage regimen optimization (frequency, length of treatment) to reach the best clinical outcome and the lowest resistant bacteria selection [73].

Pharmacokinetic/pharmacodynamic model of antibacterial agents describes the triangular correlation between potency of a drug, subject exposure to drugs, and response observed. Antimicrobial resistance emergence and selection can be viewed as an undesirable response and taken into account in the process of optimization of the drug exposure [77, 78]. There are 3 main strategies which should be used to avoid emergence of resistance.

1. Modify the exposure-response relationship to prevent emergence of resistance. Increase or decrease the exposure of bacteria to antibacterial by using different strategies like use of combination therapy, sequential therapy, and changing duration of therapy [73]. These approaches are not recommended in veterinary medicine for treatment of food producing animals regarding the regulations about drug residue. They should be applied for treatments of companion animals and horses.
2. To optimize the dose between the intervals of antimicrobial therapies through PK/PD principles, model based dosing provides patient and pathogen specific treatment. Such approach may facilitate reducing the overuse of antibacterial agents and patient exposure to needless therapy, thus serving to lessen emergence of antibacterial-resistant pathogens. PK/PD model dosing strategies are pathogen specific; they have the potential to make antibacterial therapy safer and more successful for adjustment of factors such as kidney function, primary pathogen, and limited patterns of resistance [79]. This approach used in human medicine requires extensive laboratory capacity and knowledge and should be used only in certain veterinary context such as treatment of highly valuable animals (e.g., horses).
3. Give consideration to use correct dose of drug to suppress the amplification of less susceptible mutant bacterial subpopulation. Moreover PK/PD can analyze the shape of mutant selection window (MSW) for resistance prediction [80]. This approach must be followed for veterinary drugs and was applied for few new veterinary drugs [81].

Earlier studies indicated different PK/PD model parameters used for the prevention of resistance. As in the research on neutropenic rat infection model with fluoroquinolone treatment used for* Pseudomonas aeruginosa* infection, Drusano et al. [82] found the peak to be best linked to survival when the peak/MIC ratio was above 10, while the AUC was best correlated with survival when the peak/MIC ratio was below 10 [82, 83]. Further, a larger AUC/MIC or peak/MIC ratio is generally needed to prevent resistant bacteria [83]. For the concentration-dependent antibiotics it is the best parameter related to prevent from resistance. In connection with this investigation the increased level of the drug at least 8 to 10 times of the MIC prevents the emergence of resistance subpopulation, which could be accomplished by using daily dose of aminoglycosides, or using the most potent fluoroquinolones, or high dose of *β*-lactam. Some investigators used the animal infection model and* in vitro* study to determine the C_max_/MIC and AUC/MIC ratio for fluoroquinolones and suggested that AUC/MIC is an important parameter to be considered for the prevention of resistance emergence. Some researchers suggested that AUC/MIC is important in the prevention of the emergence of resistance in fluoroquinolones [84, 85]. In patient of nosocomial acute respiratory tract infection treated with antimicrobial within AUC/MIC values <100, about 40% of patient showed stepwise increases in MIC by day 4, and about 80% by day 20 showed reduced susceptibility, whereas with an AUC/MIC >100, only 8% of pathogens developed resistance by 20 days after initiation of therapy [86]. Some researchers investigated that increased exposure to antimicrobial agents is best to prevent from resistance [87].

The mutant prevention concentration (MPC) is a new interesting concept for trying to minimize the emergence of resistance [88]. Antibacterial dosing that yields concentration during the whole dosing interval above the MPC would prevent from bacterial resistance. This may possibly be achieved by shorter dosing interval and using high doses as shown in Figure 2 decreases the time within the MSW, by using compound with smaller difference between MIC and MPC [71]. The MPC idea is derived from the hypothesis of “mutant selection window” (MSW), which postulates that a specific concentration of drug exists where antibiotic exposure selects for bacterial mutant strains with reduced antibacterial susceptibility. In mutant selection window the lowest concentration is the lower boundary that exerts selective pressure and inhibits bacterial colony formation by 99% (MIC). According to this assumption, the probable cause for clinical failures is that the concentration of drugs falls within the MSW* in vivo* so that resistant mutants are enriched, with associated loss in susceptibility. In one investigation the animals were treated with fluoroquinolone, and the drug concentration declines from *C*_max_ to concentration below which even the wild type bacteria are unaffected. At that period first-step mutant microbes have a selective advantage over the wild type, during which the population of these mutants increases. As this population grows so does the probability of the second mutation occurring and thus the selection of double mutants which are fully resistant. The concentration which inhibits the first-step mutants is defined as the mutant prevention concentration [38]. The MPC is the lowest concentration of drug that prevents the least susceptible first step resistant mutants' growth. Pharmacokinetics may play a significant role in the use of hypothesis of MSW to slow the resistance development [89]. Challenged by the appearance and widening of numerous resistance strains worldwide, the concepts of MPC and MSW provided new conceptual basis for the approaches of PK/PD in deciding dosing guidelines: time should be maximized by treatment during which concentrations of enrofloxacin were above the MPC at the site of infection, and time was minimized during which these concentrations were in the MSW. Hence *T* > MPC, instead of *T* > MIC, was considered as the most significant aspect in the design of dosage regimens to prevent mutant selection for antimicrobial drug in aquaculture [90]. A good predictor of the selection of antimicrobial resistance is the ratio between the mutant prevention concentration (MPC) and AUC_0–24_ [91]. Cui et al. [92] showed that a value of AUC_0–24_/MPC above 25 h restricts the acquisition of resistances in a* Staphylococcus aureus* infection (Gram-positive bacterium). Other works performed* in vitro* and* in vivo* study (rabbit model) demonstrated that a ratio AUC_0–24_/MPC > 22 h and >20 h prevents resistance selection in the case of* Escherichia coli* infection (Gram-negative bacteria), respectively [67, 93, 94]. So it is clear that *T* > MPC and reduced time in mutant selection window are important considerations for minimization of antibacterial resistance.

Semimechanistic PK/PD model is also useful to investigate the effect of the antimicrobial exposure on the development of antimicrobial resistance according to time. Equations take into account the differences of effect of the drug on the susceptible and resistant bacteria which are proposed and these models can take into account the complex relationship between them. A recent work about the use of colistin methanesulfonate on* Pseudomonas aeruginosa* is a good example of this kind of model [24]. Moreover, the model can integrate pharmacokinetic variability through population pharmacokinetic modeling to study the range of bacterial outcomes. The dynamics of bacterial response will be function to their susceptibility and through this mathematical model it is also feasible to study the selection of less susceptible or resistant subpopulations [95].

## 8. PK/PD and Development of New Formulations and Drugs for Animal Uses

For the development of new antimicrobial drug in human medicine, preclinical studies based on* in vitro* studies and laboratory animal's experiments are crucial to explore its potency and establish a dose range for the first human studies. Investigation about the safety, toxicity, pharmacokinetics, and metabolism in humans is important parameters to investigate before clinical trials in humans. Safety and efficacy of the drugs will be the major drivers in the development of human drugs. The risk of development of antimicrobial resistance for the community is a new constraint recently investigated in the development. For veterinary drugs used in food producing animals, consumer safety which is the most important driver in the development of the risk residue assessment through toxicological and residue studies was the most important constraint for the development of new veterinary drugs by pharmaceutical companies. With the awareness of development of antimicrobial resistance in pathogens for human and debates about the contribution of antimicrobial veterinary exposure on the global development of antimicrobial resistance, new data requests were established by regulatory agencies to a priori assess the risk. From a food safety perspective, risk of development of antimicrobial resistance in zoonotic bacteria (*Salmonella enterica*,* Campylobacter *sp.) and commensal bacterial species (*E. coli*,* Enterococcus* sp.) is requested and must be taken into account in the optimization of the dosage regimen. So two goals for antimicrobial resistance selection prevention can be assigned for dosage regimen optimization as discussed by Toutain et al. [62]: (1) optimize the dosage regimen to reach the pathogen at the site of infection and (2) limit the exposure of the commensal flora mainly in the intestinal lumen of the treated subject. Few works have been done to study simultaneously these two objectives and demonstrate the need to develop rapid diagnostic of infection and early treatment of animals with an optimal dose, sufficient to reach the site of infection and with a low exposure of the commensal flora [96].

Drug development which is based on model has been predictable by pharmaceutical companies, regulatory agencies, and academia as a standard to renovate research of drug through the quantification of risk and combination of information from different resources across time. Pharmacokinetic (PK)/pharmacodynamic (PD) analysis plays an important role in development of drug and clinical pharmacotherapy of diverse kind of drugs [97–100]. Once the potency and spectrum of an antibacterial agent have been established by susceptibility tests, the tools and endpoints of pharmacodynamics can be used in additional investigation of the antibacterial activity and clinical potential of a new drug. Pharmacodynamics studies will help to identify whether a new antibacterial agent is bacteriostatic or bactericidal drug, a concentration-dependent killer, or time-dependent bactericidal agent. In addition, pharmacodynamic studies can clearly define the pharmacodynamics parameters that are linked to clinical efficacy and identify the minimum target specifically essential to optimize clinical efficacy. On the whole, these data help out to focus on clinical trials by assisting in the optimum dose selection. In order to develop latest antibacterial drug, the important subject is how to apply pharmacodynamic experiments to study the drug and (1) verify which parameter mainly influences clinical efficacy, (2) identify the exact drug concentration to reach at the target site of infection for better efficacy with minimal impact on commensals, and (3) use this data to express dose range for clinical trials. The efficiency of PK/PD information is certain in the development of new antimicrobials, the design of optimal dosage strategies, the more precise selection of suitable antimicrobials from formularies, and the decrease in the selection of antimicrobial resistance [101, 102]. However, the PK/PD principle is important to be used for developing dosage regimen to rejuvenate old antimicrobial agent. Thus to increase the sustainable use of antibiotic, it is important to use both healthy and diseased model for the optimization of dose and evaluation of side effect in drug development process [71]. In future drug development, other strategies to prevent resistance development should also be included such as the development of ecological model linking different levels of complexity [103]. One such strategy should be to identify the reason of resistance and to select antibiotic for which resistance mutations are rare.

## 9. Conclusion

Future work must focus on understanding the pharmacokinetics and pharmacodynamics of veterinary drugs. Such a perceptive should eventually permit the progress of new modeling approaches for dose optimization to minimize the resistance. PK/PD will provide the information related to the effect of different concentration and observed therapeutic and side effects for different kind of treatment in different animal species. In the meantime, we hate to promote the best animal husbandry practices to prevent infection and limit antimicrobial drug use for therapeutic targeted treatments. A comprehensive systematic review at this time would include additional knowledge of PK/PD model not covered in previous reviews and inform clinical decision making for patients, clinicians, health systems, and stakeholders. Previous reviews have not determined the impact of using these measures on the outcomes outlined above. Here are concerns with the purpose of rising resistance with bacteria, when left untreated, which could go up to the peak at which effectiveness of several of the mainly important drugs will no longer be expected and several bacterial infections might once again turn into untreatable [60].

This review supported that PK-PD modeling is a powerful tool in veterinary field; it explains the drugs effect against microorganism and therefore it is suggested to be used in preclinical and clinical development of veterinary drugs to optimize dosing strategy. To be adaptable for use in veterinary clinical practice, the strategy will however be adapted to integrate the public health outcomes which are the development resistance in zoonotic and commensals bacteria. Pharmacokinetics and pharmacodynamics approaches should be adapted. This would be possible by the cooperation of clinical veterinary pharmacologist and microbiologist with clinical practitioner to optimize dose and treatment duration.

The training of clinical practitioner to understand the PK/PD approach is also required to develop a more tactical prescription and use of drugs. So PK/PD integration of antimicrobial agents for veterinary research provides a chance for the worthy progress towards dosage optimization (and minimizing) the use of chemotherapeutics agents in animals. Considering that antibacterial treatment is our primary and in many cases the only way to treat infection, more detailed studies based on PK/PD model in veterinary medicine are crucial to our future ability to infection in animals.

## Figures and Tables

**Table 1:** General classification of antimicrobial drugs as a concentration- and time-dependent activity.

Time-dependent	Concentration-dependent	Codependent
Beta-lactams	Aminoglycosides	Beta/lactams
Macrolides (except azithromycin)	Fluoroquinolones	Fluoroquinolones
Clindamycin	Metronidazole	Glycopeptides
Vancomycin	Colistin, rifamycins	Tetracycline

**Table 2:** Definition of important PK, PD, and PK/PD indices.

PK/PD index	Definition	Unit	References
Pharmacodynamics			
MIC	The minimal inhibitory concentration is defined as the lowest concentration of antibiotic that inhibits completely the growth of the specific organism being tested.	mg/L or *µ*g/mL	Mouton et al., 2005 [34]
MBC	MBC is the lowest concentration at which 99.9% reduction in bacterial count is achieved	mg/L or *µ*g/mL	Tayler et al., 1983 [44]
MPC	MPC (mutant prevention concentration): the lowest concentration that prevents the emergence of mutants after 120 hours of incubation	mg/L or *µ*g/mL	Shimizu et al., 2013 [45]
PAE	Postantibiotic effect is the time of suppression of bacterial growth after the bacteria are exposed to antibacterial for a short time	Time (h)	Mouton et al., 2005 [34]

Pharmacokinetics			
AUC	The area under the concentration time curve over 24 h at steady state unless otherwise stated. It is equivalent to a single dose AUC_0-∞_	*µ*g·h/mL	Mouton et al., 2005 [34]
*f*	Prefix indicating that the pharmacokinetic parameter values or PK/PD index values used are unbound (free) fractions of the drug		
*C*_Max⁡_	The highest concentration of drug reached or estimated in the compartment of reference	mg/L or *µ*g/mL	Mouton et al., 2005 [34]

PK/PD integration			
*T* > MIC	The cumulative percentage of 24 h period in which the drug concentration exceeds the MIC at steady state pharmacokinetic condition	%	Mouton et al., 2005 [34]
AUC/MIC	The area under the concentration time curve divided by MIC	No unit	Mouton et al., 2005 [34]
*C*_Max⁡_/MIC	The peak concentration of drug divided by MIC	No unit	Mouton et al., 2005 [34]

**Table 3:** Classification of antibacterial drugs according to pharmacokinetics and pharmacodynamics indices. Different group of antibacterials, their bacterial effect, and PK/PD integration most closely related to their clinical effect.

Group	Drugs	PK/PD indices	Activity	Bacterial effect	Duration of PAE	References
1	Aminoglycosides	*C*_Max⁡_/MIC or AUC/MIC	Primarily bactericidal	Concentration-dependent	Prolonged	Martinez et al., 2014 [17]
	Fluoroquinolone	AUC/MIC	Bactericidal	Concentration-dependent	Prolonged	Martinez et al., 2014 [17]
	Enrofloxacin	*C*_peak_/MIC/AUC : MIC	Bacteriostatic bactericidal	Concentration-dependent		Balaje et al., 2013 [49]
	Azithromycin	AUC_24_/MIC				
	Tetracycline	AUC_24_/MIC	Bacteriostatic	Time-dependent	Prolonged	Martinez et al., 2014 [17]
	Colistin	AUC/MIC		Concentration-dependent	Short	Hengzhuang et al., 2012 [59]
	Metronidazole	*C*_peak_/MIC/AUC : MIC		Concentration-dependent		Paul et al., 2005 [60]

2	Ketolides	%*T* > MIC	Bacteriostatic or bactericidal	Time-dependent	Prolonged	Martinez et al., 2014 [17]
	Penicillins Carbapenems Cephalosporins	%*T* > MIC	Bactericidal	Time-dependent	Non or brief against Gram-negative and prolonged against Gram-positive	Martinez et al., 2014 [17]
	Lincosamides (clindamycin)	%*T* > MIC	Bacteriostatic	Time-dependent	Brief	Martinez et al., 2014 [17]
	Trimethoprim	%*T* > MIC	Bacteriostatic alone and bactericidal with combination	Time-dependent	Brief	Martinez et al., 2014 [17]
	Glycopeptides (vancomycin)	%*T* > MIC	Bactericidal	Time-dependent	Prolonged	Martinez et al., 2014 [17]
