# Corrigendum to “Application of PK/PD Modeling in Veterinary Field: Dose Optimization and Drug Resistance Prediction”

**DOI:** 10.1155/2017/1408737

**Published:** 2017-11-06

**Authors:** Ijaz Ahmad, Lingli Huang, Haihong Hao, Pascal Sanders, Zonghui Yuan

**Affiliations:** ^1^National Reference Laboratory of Veterinary Drug Residues (HZAU) and MAO Key Laboratory for Detection of Veterinary Drug Residues, Huazhong Agricultural University, Wuhan, Hubei 430070, China; ^2^The University of Agriculture Peshawar, Khyber Pakhtunkhwa 25130, Pakistan; ^3^MOA Laboratory for Risk Assessment of Quality and Safety of Livestock and Poultry Products, Huazhong Agricultural University, Wuhan, Hubei 430070, China; ^4^Laboratory of Fougères, French Agency for Food, Environmental and Occupational Safety, 94701 Maisons-Alfort Cedex, France

In the article titled “Application of PK/PD Modeling in Veterinary Field: Dose Optimization and Drug Resistance Prediction” [1], there was an error in Table 3. Some antibiotic groups were not correctly classified in Table 3 as raised by Benini and Fumagalli in [2]. The correct statement is that ketolides exhibit concentration-dependent killing and have prolonged persistence and the PK/PD indices responsible for efficacy are AUC24/MIC. Clindamycin and vancomycin exhibit time-dependent killing and have moderate to prolonged persistence and PK/PD indices responsible for efficacy are AUC24/MIC. The corrected table is shown as follows (see Table 3).

## Figures and Tables

**Table 3:** Classification of antibacterial drugs according to pharmacokinetics and pharmacodynamics indices: different groups of antibacterials, their bacterial effect, and PK/PD integration most closely related their clinical effect.

Group	Drugs	PK/PD indices	Activity	Bacterial effect	Duration of PAE	References
1	Aminoglycosides	*C*_max_/MIC or AUC/MIC	Primarily bactericidal	Concentration-dependent	Prolonged	Martinez et al., 2014 [17]
	Fluoroquinolone	AUC/MIC	Bactericidal	Concentration-dependent	Prolonged	Martinez et al., 2014 [17]
	Enrofloxacin e	*C*_peak_/MIC/AUC : MIC	Bacteriostatic bactericidal	Concentration-dependent		Balaje et al., 2013 [49]
	Azithromycin	AUC_24_/MIC				
	Tetracycline	AUC_24_/MIC	Bacteriostatic	Time-dependent	Prolonged	Martinez et al., 2014 [17]
	Colistin	AUC/MIC		Concentration-dependent	Short	Hengzhuang et al., 2012 [59]
	Metronidazole	*C*_peak_/MIC/AUC : MIC		Concentration-dependent		Paul et al., 2005 [60]

2	Ketolides	AUC/MIC	Bacteriostatic or bactericidal	Concentration-dependent	Prolonged	Martinez et al., 2014 [17]
	Penicillins Carbapenems Cephalosporins	%*T* > MIC	Bactericidal	Time-dependent	Non or brief against Gram-negative and prolonged against Gram-positive	Martinez et al., 2014 [17]
	Lincosamides (clindamycin)	AUC/MIC	Bacteriostatic	Time-dependent	Brief	Martinez et al., 2014 [17]
	Trimethoprim	%*T* > MIC	Bacteriostatic alone and bactericidal with combination	Time-dependent	Brief	Martinez et al., 2014 [17]
	Glycopeptides (vancomycin)	AUC/MIC	Bactericidal	Time-dependent	Prolonged	Martinez et al., 2014 [17]
